# Metabolomics analysis of cerebrospinal fluid suggests citric acid cycle aberrations in bipolar disorder

**DOI:** 10.1016/j.nsa.2022.100108

**Published:** 2022-06-30

**Authors:** Erik Smedler, Alireza M. Salehi, Aurimantas Pelanis, Ana Andreazza, Erik Pålsson, Timea Sparding, Mikael Landén

**Affiliations:** a Department of Neuroscience and Physiology, Sahlgrenska Academy at Gothenburg University, Gothenburg, Sweden; b Department of Medical Epidemiology and Biostatistics, Karolinska Institutet, Stockholm, Sweden; c RISE, Research Institutes of Sweden, Digital Health, Stockholm, Sweden; d Department of Pharmacology and Toxicology, University of Toronto, Canada

**Keywords:** MeSH): bipolar disorder, Metabolome, Citric acid cycle, Cerebrospinal fluid

## Abstract

Mounting evidence indicates mitochondrial dysfunction in bipolar disorder pathophysiology. Here, we employed Proton Nuclear Magnetic Resonance Spectroscopy (^1^H NMR) of cerebrospinal fluid (CSF) samples from well-characterized bipolar disorder patients (n ​= ​67) and healthy controls (n ​= ​55) in order to measure absolute concentrations of multiple metabolites. Focusing on four citric acid cycle metabolites — citrate, glucose, lactate, and pyruvate — we found higher concentrations of both citrate and glucose in patients compared with controls after correcting for age, sex and body mass index, but only the difference in CSF citrate survived correction for multiple comparisons. Within the patient group, CSF citrate concentrations were higher among lithium users than non-users. In conclusion, this report adds further evidence for a mitochondrial dysfunction in bipolar disorder.

## 1 Introduction

Bipolar disorder is a severe psychiatric disease characterized by episodes of depression and mania/hypomania affecting around 1–2.4% of the population depending on definition (Kessler et al., 2005; Merikangas et al., 2011). Although several studies on twins and families show the importance of genetic factors, and genome-wide association studies have identified 64 common genetic risk variants (Mullins et al., 2021), the precise pathophysiology of bipolar disorder remains unknown.

One line of evidence implicates mitochondrial dysfunction in the pathogenesis, which has prompted the mitochondrial dysfunction hypothesis (Andreazza et al., 2018; Kato, 2011). This hypothesis is based on several lines of evidence, including genetic analyses of *post mortem* tissue indicating changes in the transcription of mitochondria related genes (Iwamoto et al., 2005; Konradi et al., 2004; Scola et al., 2013; Sun et al., 2006), and the fact that mood-stabilizing agents such as lithium and valproate influence mitochondrial function (Bachmann et al., 2009; Osete et al., 2021b).

The citric acid cycle is a series of chemical reactions that occurs in the mitochondria and serves to provide the electron transport chain with substrate, by oxidating acetyl coenzyme A derived from proteins, fat, and carbohydrates. Via oxidative phosphorylation, the electron transport chain subsequently produces ATP (or less energy via lactate in case of anaerobic respiration). We and others have previously found evidence of aberrations in oxidative phosphorylation (Stork and Renshaw, 2005) in general and in the citric acid cycle in specific in bipolar disorder. First, we found lower concentrations of citrate and higher concentrations of pyruvate in serum from bipolar disorder patients as compared with controls (Yoshimi et al., 2016b). Second, in the same cohort we found higher isocitrate concentrations in cerebrospinal fluid (CSF) from bipolar disorder patients compared with controls (Yoshimi et al., 2016a). Also, a large number of magnetic resonance spectroscopy studies in bipolar disorder patients have revealed aberrations in multiple metabolic pathways of the mitochondria, including oxidative phosphorylation and phospholipid metabolism (Dogan et al., 2018; Kuang et al., 2018; Stork and Renshaw, 2005). Together, these separate studies can be collected in a theory of mitochondrial dysfunction in bipolar disorder encompassing increased concentrations of lactate, glutamate, choline and decreased levels of N-acetyl aspartate.

The metabolome comprises all metabolites found in a sample, and metabolomics is the study of the metabolome, i.e., small molecules that are intermediates or products of metabolism (Roberts et al., 2011). Both mass spectrometry (MS) and nuclear magnetic resonance (NMR) spectroscopy can be deployed to identify metabolites related to disease (Holmes et al., 2008). The advantage of NMR spectroscopy is that it can be used to quantitatively determine concentrations of metabolites with high concentration in biofluids without any separation process in comparison with MS, and do not require any extra sample preparation (Emwas, 2015).

The aim of this metabolomic study was to replicate the finding of altered function of the citric acid cycle in bipolar disorder in a new case-control cohort independent from our previous studies. To this end, we used ^1^H NMR spectroscopy to measure 44 metabolites in CSF from persons with bipolar disorder and healthy controls.

## 2 Materials and methods

### 2.1 Participants

The study sample included 67 patients with bipolar disorder and 55 healthy controls. Details on exclusion and inclusion criteria, diagnostic tools, and methods can be found elsewhere (Rolstad et al., 2015). Briefly, patients were recruited from the Gothenburg arm of the St. Göran Bipolar Project, which enrols patients from the bipolar unit at the Affective Clinic, Gothenburg, Sweden. A Swedish version of the Affective Disorder Evaluation (ADE) was used for clinical assessment (Sachs et al., 2003). Patients were diagnosed according to DSM-IV criteria as per the Structured Clinical Interview for DSM-IV (SCID) included in the ADE. The inclusion criteria for this report were age ≥18 years, and a DSM-IV bipolar spectrum diagnosis (type 1, type 2, not otherwise specified). Participants with bipolar disorder were also interviewed using the M.I.N.I. International Neuropsychiatric Interview (M.I.N.I.) to screen for comorbid psychiatric diagnoses.

Healthy controls were recruited from the general population through Statistics Sweden (www.scb.se) that randomly identified individuals living in the same catchment area as patients. Those who responded to a letter informing about the study were first interviewed over phone by research nurses and screened for the exclusion criteria: any current psychiatric disorder or any current use of psychotropic drugs, bipolar disorder or schizophrenia in first degree relatives, neurological diseases (excluding mild migraine), substance abuse, untreated endocrine disorders, or pregnancy. Eligible individuals were scheduled for a physical visit where M.I.N.I. and selected parts of the ADE were used to screen for psychiatric morbidity.

The study was approved by the Regional Ethics Committee in Stockholm and conducted in accordance with the Helsinki Protocol. After a complete description of the study, all enrolled patients and controls consented orally and in writing to participate in the study. Healthy controls but not persons with bipolar disorder were reimbursed for participating in the study.

### 2.2 CSF sampling

CSF sampling by means of lumbar puncture occurred when the bipolar disorder participants were in a stable mood, i.e., they were not suffering from an acute mood episode according to the treating physician. Sampling occurred in the morning after an overnight fast. The spinal needle was inserted into the L3/L4 or L4/L5 interspace and a standardized volume of 12 ​ml CSF was collected in a polypropylene tube, gently inverted to avoid gradient effects, centrifuged for 10 ​min (1.8x1000 rcf) and aliquoted into 0.5 ​ml polypropylene tubes. The aliquoted CSF samples were stored at −80 ​°C at the Karolinska Institutet Biobank, Stockholm, Sweden pending analysis. The collection of CSF in controls followed an identical procedure.

#### 2.2.1 Metabolic profiling

For ^1^H NMR analysis, the CSF samples were thawed and 230 ​μl of CSF was transferred from the biobank vials to cryovials. The following steps were performed using a Bruker SamplePro liquid handling system: 200 ​μl aliquots were mixed with 400 ​μl of buffer [Polybutylene succinate (PBS) with 10% D_2_O, NaN_3_ and 5.5 ​mM trimethylsilylpropanoic acid (TMSP), pH 7.4] before transfer to 5 ​mm SampleJet tubes. Mixing was performed directly in the SampleJet tubes using a 2-step mixing cycle. The proton spectra were collected at 37 ​°C on a Bruker 600 ​MHz Avance III HD spectrometer equipped with a 5 ​mm TCI cryo-probe. T2-relaxation-edited spectra were recorded with a total cpmg spin-lock time of 100 ​ms to attenuate broad signals from proteins and lipids. 64 scans and 64k data points were recorded with a spectral width of 14 ​ppm and a relaxation delay of 1.1 ​s. The free induction decay (FID) was zero-filled, and an exponential line-broadening function of 0.3 ​Hz was applied to the FID prior to Fourier transformation. Processing was performed in Topspin 3.1 (Bruker Biospin, Rheinstetten, Germany).

CSF metabolites were assigned by comparison with chemical shift. All spectra were manually phased and calibrated using the TMSP resonance at 0 ​ppm. In order to exploit all metabolic information embedded in the spectra, all NMR spectra (0.1–10 ​ppm) were segmented into equal widths of both 0.01 ​ppm and 0.0015 ​ppm using the AMIX package (Bruker Biospin, Germany). Spectral regions of δ4.5–5.2 were removed in order to eliminate variations caused by imperfect water suppression. The remaining spectral segments in each NMR spectrum were normalized to the total sum of the spectral intensity to partially compensate for differences in concentration among the numerous metabolites. In addition, metabolites were quantified using NMR suite 8.0 (www.chenomx.com).

For both practical and experimental reasons, we decided to measure 44 metabolites (citrate 2-oxoisocaproate, histidine, glucose, serine, formate, lactate, dimethyl sulfone, creatinine, ascorbate, ornithine, glutamine, 3-hydroxyisobutyrate, pyruvate, 3-methyl-2-oxovalerate, phenylalanin, arginine, hypoxanthine, dimethylamine, 3-hydroxybutyrate, pyroglutamate, alanine, mannose, ethanolamine, acetone, fructose, tyrosine, isoleucine, threonine, acetoacetate, acetate, methanol, myo-inositol, lysine, choline, propylene glycol, creatine, ethanol, leucine, 3-hydroxyisovalerate, 2-hydroxyisovalerate, 2-hydroxybutyrate, valine, and arabinitol), spanning major metabolic pathways (e.g. amino acid, fatty acid nucleic acid and ketone metabolism). However, in the statistical regression analyses we focused on four metabolites related to the citric acid cycle (citrate, glucose, lactate, and pyruvate). We measured citrate instead of isocitrate, as it is more readily detected ^1^H NMR.

### 2.3 Statistical analysis

All analyses were performed using custom made scripts in MATLAB (MathWorks R2021b). Hypothesis testing was done using unpaired Student's *t*-test or Wilcoxon rank-sum test for numerical data and Fisher's exact test for dichotomous data. Numeric values are presented as means and standard deviation or as 95% confidence intervals with unadjusted p values. Regression analyses were performed using multiple linear regression with the metabolite concentration as dependent variable. Pairwise correlations were calculated using Pearson correlations (*corrcoef*).

## 3 Results

This study includes 67 patients with bipolar disorder and 55 healthy controls. There were no differences between the groups with regards to sex, age, body mass index (BMI), proportion of smokers or current illicit drug use (Table 1). Using ^1^H NMR to analyse CSF samples, we successfully identified the concentration for 44 metabolites spanning metabolism of carbohydrates, proteins and lipids (**Supplementary data**). To test the hypothesis of aberrations in citric acid cycle in bipolar disorder, we modeled the concentrations of citrate, glucose, lactate and pyruvate using multiple linear regression with the independent variables case/control, sex, BMI, and age.

**Table 1:** Characteristics of the participants. BMI: Body Mass Index. GAF: Global Assessment of Functioning. ^1^ 1 missing. ^#^ Fisher's test. ^§^ Wilcoxon rank-sum test. Age, BMI and GAF are presented with mean values and standard deviation.

	Patients	Controls	P value
N	67	55	
Females (%)	64.2	54.6	0.35^#^
Age	39.3 (12.6)	43.1 (12)	0.087^§^
BMI	26.1 (7.9)	24.8 (3.6)	0.063^§^
Smoking (%)	28.4	14.6	0.082^#^
Current illicit drug use (%)	6	0^1^	0.13^#^
GAF	59.2 (9.1)^1^	88.7 (7.1)	<0.001^§^

The CSF concentrations of citrate (mean, 95% confidence interval: 61.9, [57.5, 66.2] μM vs 54.8, [51.7, 57.8] μM, p ​= ​0.0028) and glucose (750.6, [709.9, 791.3] μM vs 700.5, [679.5, 722.5] μM, p ​= ​0.026) were higher in cases than controls (see **Supplementary data** for complete results). The difference for citrate, but not glucose, remained significant after Bonferroni correction (α_Bonferroni_ ​= ​0.05/4 ​= ​0.0125). The CSF concentration of lactate (473, [451, 494] μM vs 447, [430, 464] μM, p ​= ​0.08) and pyruvate (20.2, [18.7, 21.8] μM vs 18.9, [17.4, 20.4] μM, p ​= ​0.21) did not differ significantly between cases and controls. As the CSF samples were diluted 1:3, the glucose and lactate concentrations correspond to approximately 2.8 ​mM and 1.8 ​mM, respectively (Leen et al., 2012). All four metabolites had strong and significant intercorrelations for the entire group of subjects (patients and controls, **Supplementary data**).

**Fig. 1:**
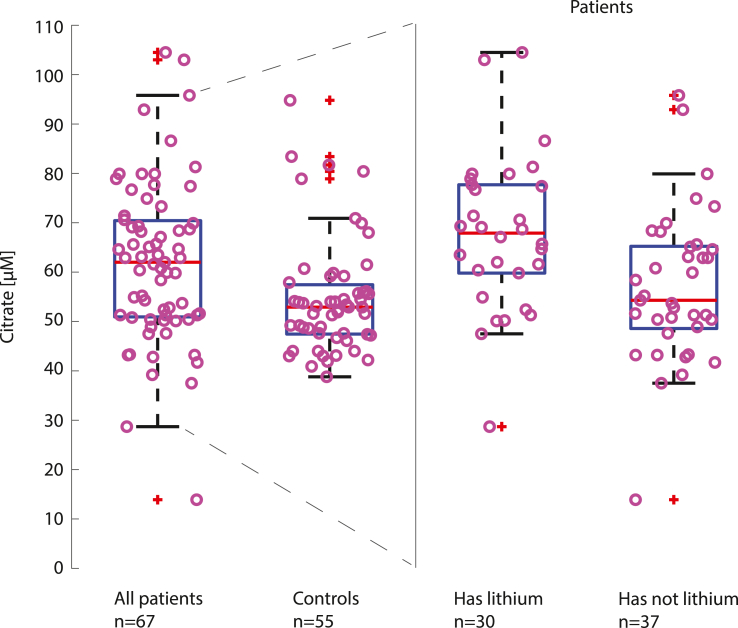
CSF concentration of citrate for all patients, controls, patients with lithium treatment at those without lithium treatment.

Next, we sought to investigate if the difference in metabolite levels between patients and controls was dependent on medication status. However, as only patients were exposed to psychotropic medications, we used multiple linear regression with medication status as independent variables (treatment with lithium, valproate, or lamotrigine) in patients only (Table 2). Lithium explained most of the variance regarding citrate (β ​= ​10.4, see **Supplementary data** for complete results). Moreover, in cases only, the concentration of citrate was significantly higher in those with lithium treatment (n ​= ​30) compared to those without (n ​= ​37, 67.8, [62.2, 73.5] μM vs 57, [52, 62] μM, p ​= ​0.0065 Fig. 1).

**Table 2:** Patient specific characteristics.

	N	%
*Diagnosis*		
BD type 1	25	37.3
BD type 2	36	53.7
BD NOS	6	9
*Clinical data*		
Any mood-stabilizer	47	70.2
Lithium	30	44.8
Valproate	10	14.9
Lamotrigine	28	41.8
Carbamazepine	4	6
Antidepressant	38	56.7
Antipsychotic	22	32.8
Previous suicide attempt	24	35.8
History of psychosis	16	23.9

Abbreviations: BD: Bipolar Disorder. NOS: Not Otherwise Specified.

## 4 Discussion

In this cross-sectional cohort study of bipolar disorder patients and healthy controls, we analyzed four CSF metabolites related to the citric acid cycle using ^1^H NMR: citrate, glucose, lactate and pyruvate. We found that the CSF concentrations of citrate was on average 13% higher in cases than controls, and that the difference remained after adjusting for age, sex, and BMI as well as multiple comparisons. In cases only, CSF citrate was 19% higher among those treated with lithium.

We have previously used mass spectrometry to analyse metabolites and reported higher CSF concentration of isocitrate (Yoshimi et al., 2016a). Here, we reproduce changes in the citric acid cycle in bipolar disorder in an independent cohort of patients and controls using a different metabolomic method. However, isocitrate is more readily detected with mass spectrometry than ^1^H NMR (thus not measured here), and here we detect higher CSF concentration of citrate in bipolar disorder than controls. Citrate and coenzyme A is the result of citrate synthase catalysing the reaction between oxaloacetate and acetyl-coenzyme A. Interestingly, one previous paper measured citrate synthase activity in platelets from bipolar and unipolar depressed patients and found lower activity as compared to healthy controls (Zverova et al., 2019). Next, citrate is converted to isocitrate via isomerization by aconitate hydratase. In contrast to this study, we previously reported higher levels of pyruvate in CSF from BD patients (Yoshimi et al., 2016a). As in another previous study, we also now report higher levels of CSF glucose (Regenold et al., 2009) as well as strong correlation between CSF glucose and lactate levels (r ​= ​0.74, p ​= ​4.5∗10^−22^).

One of the first piece of evidence for the involvement of mitochondrial function in BD is lower intracellular pH in the brain of patients (Kato et al., 1992), likely due to increased levels of lactate (Dogan et al., 2018; Kuang et al., 2018; Stork and Renshaw, 2005). Here we also found numerically higher levels of lactate in patients than controls, albeit not significant (adjusted p ​= ​0.07). Interestingly, intracellular pH in bipolar disorder appears to be state dependent, as patients in both manic and depressive episodes have less acidic levels (Kato et al., 1993). Also, this finding is unlikely to be due to lithium treatment as even drug-free patients have lower pH (Kato et al., 1998). Several studies have investigated the effect of pharamcotherapy (especially lithium) on the various results on metabolites with inconclusive results (Lundberg et al., 2020; Ochoa, 2022). In our previous studies, medication status did not affect the level of citrate or isocitrate in patients (Yoshimi et al., 2016a). But in the present study, CSF citrate was higher among patients that used lithium. Given that no control subject used lithium, it was not possible to adjust for lithium use. Our study design can hence not disentangle whether lithium caused higher CSF citrate concentrations or identifies a subgroup of bipolar disorder patients. In our previous rat models, chronic treatment with lithium increased the serum concentration of citrate, but not isocitrate in CSF (Yoshimi et al., 2016a, 2016b). Lithium may reverse the inhibition of citrate synthase in hippocampus, as shown in one mania model in rats (Correa et al., 2007).

Citrate is converted to isocitrate via isomerization by aconitate hydratase. By analyzing mRNA levels in *post mortem* tissue from prefrontal cortex of bipolar patients and controls, we previously reported lower levels of *IDH3A* and *IDH3B*, but equal levels of *ACO* (Yoshimi et al., 2016a). These genes encode enzymes that convert isocitrate to α-ketoglutarate (isocitrate dehydrogenase) and citrate to isocitrate (aconitate hydratase), respectively. Thus, lower enzymatic activity of IDH3 would lead to increased levels of isocitrate and subsequently citrate, as reported here. On the contrary, another paper reports no difference on protein level of IDH3 in *post mortem* prefrontal cortex (Scola et al., 2018). Further, citrate may not only be seen as a marker of the citric acid cycle, but could also convey information from the mitochondria to the rest of cell and potentially the extracellular space (Martinez-Reyes and Chandel, 2020). Upon exiting the mitochondria, citrate can be converted back to acetyl-CoA, that in turn may regulate chromatin dynamics during differentiation and growth factor stimulation (Wellen et al., 2009).

Strengths of this study include the novel large CSF collection from well-characterized bipolar disorder cases and controls, which allowed us to replicate previous findings in an independent cohort by means of a different metabolomic technique. As patients are considered euthymic at the timing of lumbar punctue, results can be seen as trait markers. A limitation of this naturalistic cohort study is that patients were exposed to pharmacotherapies that vary depending on clinical needs, which cannot be controlled for statistically as no controls were exposed to psychiatric medications. Hence, the present study cannot clarify if altered levels of CSF citrate is a feature of the bipolar disorder pathophysiology or an effect of lithium treatment. Finally, to fully investigate all potential aberrations in the citric acid cycle, one would preferably measure multiple metabolites in addition to those measure here.

However, the fact that mitochondrial diseases often present with psychiatric symptoms suggest that mitochondrial dysfunction has a causative role (Anglin et al., 2012; Fattal et al., 2007; Inczedy-Farkas et al., 2012; Mancuso et al., 2013). Further, by using an induced pluripotent stem cell model of bipolar disorder, two studies revealed enhanced mitochondrial function in conjunction with electrical hyperactivity in hippocampal dentate gyrus-like neurons (Mertens et al., 2015; Stern et al., 2018). Interestingly, this hyperactivity could be selectively normalized in neurons derived from lithium responsive patients, while restoring mitochondrial function. Another recent study found that treatment with lithium (and partially valproate and lamotrigine) in neural progenitor cells derived from induced pluripotent stem cells from bipolar disorder patients resulted in increased mitochondrial respiration (Osete et al., 2021a).

In conclusion, we report higher levels of citrate in CSF of bipolar disorder patients compared with healthy controls. This may reflect either increased mitochondrial activity in certain brain regions, or being the effect of lithium treatment. Future studies including drug naïve patients, or a longitudinal study where patients start and discontinue medication, would make it possible to distinguish between correlation and causation.

## Disclosures

ML declares that he has received lecture honoraria from Lundbeck pharmaceuticals outside the present work. The other authors declare no conflict of interest.

## Role of funding source

The funding source was not involved in analysis, interpretation of data or in writing the manuscript.

## Conflict of interest

ML declares that he has received lecture honoraria from Lundbeck pharmaceuticals outside the present work. The other authors declare no conflict of interest.

## References

[bib1] Andreazza A.C., Duong A., Young L.T. (2018). Bipolar disorder as a mitochondrial disease. Biol. Psychiatr..

[bib2] Anglin R.E., Garside S.L., Tarnopolsky M.A., Mazurek M.F., Rosebush P.I. (2012). The psychiatric manifestations of mitochondrial disorders: a case and review of the literature. J. Clin. Psychiatr..

[bib3] Bachmann R.F., Wang Y., Yuan P., Zhou R., Li X., Alesci S., Du J., Manji H.K. (2009). Common effects of lithium and valproate on mitochondrial functions: protection against methamphetamine-induced mitochondrial damage. Int. J. Neuropsychopharmacol..

[bib4] Correa C., Amboni G., Assis L.C., Martins M.R., Kapczinski F., Streck E.L., Quevedo J. (2007). Effects of lithium and valproate on hippocampus citrate synthase activity in an animal model of mania. Prog. Neuro-Psychopharmacol. Biol. Psychiatry.

[bib5] Dogan A.E., Yuksel C., Du F., Chouinard V.A., Ongur D. (2018). Brain lactate and pH in schizophrenia and bipolar disorder: a systematic review of findings from magnetic resonance studies. Neuropsychopharmacology.

[bib6] Emwas A.H. (2015). The strengths and weaknesses of NMR spectroscopy and mass spectrometry with particular focus on metabolomics research. Methods Mol. Biol..

[bib7] Fattal O., Link J., Quinn K., Cohen B.H., Franco K. (2007). Psychiatric comorbidity in 36 adults with mitochondrial cytopathies. CNS Spectr..

[bib8] Holmes E., Wilson I.D., Nicholson J.K. (2008). Metabolic phenotyping in health and disease. Cell.

[bib9] Inczedy-Farkas G., Remenyi V., Gal A., Varga Z., Balla P., Udvardy-Meszaros A., Bereznai B., Molnar M.J. (2012). Psychiatric symptoms of patients with primary mitochondrial DNA disorders. Behav. Brain Funct..

[bib10] Iwamoto K., Bundo M., Kato T. (2005). Altered expression of mitochondria-related genes in postmortem brains of patients with bipolar disorder or schizophrenia, as revealed by large-scale DNA microarray analysis. Hum. Mol. Genet..

[bib11] Kato T. (2011). Mitochondrial dysfunction and bipolar disorder. Curr. Top. Behav. Neurosci..

[bib12] Kato T., Murashita J., Kamiya A., Shioiri T., Kato N., Inubushi T. (1998). Decreased brain intracellular pH measured by 31P-MRS in bipolar disorder: a confirmation in drug-free patients and correlation with white matter hyperintensity. Eur. Arch. Psychiatr. Clin. Neurosci..

[bib13] Kato T., Takahashi S., Shioiri T., Inubushi T. (1992). Brain phosphorous metabolism in depressive disorders detected by phosphorus-31 magnetic resonance spectroscopy. J. Affect. Disord..

[bib14] Kato T., Takahashi S., Shioiri T., Inubushi T. (1993). Alterations in brain phosphorous metabolism in bipolar disorder detected by in vivo 31P and 7Li magnetic resonance spectroscopy. J. Affect. Disord..

[bib15] Kessler R.C., Berglund P., Demler O., Jin R., Merikangas K.R., Walters E.E. (2005). Lifetime prevalence and age-of-onset distributions of DSM-IV disorders in the national comorbidity survey replication. Arch. Gen. Psychiatr..

[bib16] Konradi C., Eaton M., MacDonald M.L., Walsh J., Benes F.M., Heckers S. (2004). Molecular evidence for mitochondrial dysfunction in bipolar disorder. Arch. Gen. Psychiatr..

[bib17] Kuang H., Duong A., Jeong H., Zachos K., Andreazza A.C. (2018). Lactate in bipolar disorder: a systematic review and meta-analysis. Psychiatr. Clin. Neurosci..

[bib18] Leen W.G., Willemsen M.A., Wevers R.A., Verbeek M.M. (2012). Cerebrospinal fluid glucose and lactate: age-specific reference values and implications for clinical practice. PLoS One.

[bib19] Lundberg M., Millischer V., Backlund L., Martinsson L., Stenvinkel P., Sellgren C.M., Lavebratt C., Schalling M. (2020). Lithium and the interplay between telomeres and mitochondria in bipolar disorder. Front. Psychiatr..

[bib20] Mancuso M., Orsucci D., Ienco E.C., Pini E., Choub A., Siciliano G. (2013). Psychiatric involvement in adult patients with mitochondrial disease. Neurol. Sci..

[bib21] Martinez-Reyes I., Chandel N.S. (2020). Mitochondrial TCA cycle metabolites control physiology and disease. Nat. Commun..

[bib22] Merikangas K.R., Jin R., He J.P., Kessler R.C., Lee S., Sampson N.A., Viana M.C., Andrade L.H., Hu C., Karam E.G., Ladea M., Medina-Mora M.E., Ono Y., Posada-Villa J., Sagar R., Wells J.E., Zarkov Z. (2011). Prevalence and correlates of bipolar spectrum disorder in the world mental health survey initiative. Arch. Gen. Psychiatr..

[bib23] Mertens J., Wang Q.W., Kim Y., Yu D.X., Pham S., Yang B., Zheng Y., Diffenderfer K.E., Zhang J., Soltani S., Eames T., Schafer S.T., Boyer L., Marchetto M.C., Nurnberger J.I., Calabrese J.R., Odegaard K.J., McCarthy M.J., Zandi P.P., Alda M., Nievergelt C.M., Pharmacogenomics of Bipolar Disorder S., Mi S., Brennand K.J., Kelsoe J.R., Gage F.H., Yao J. (2015). Differential responses to lithium in hyperexcitable neurons from patients with bipolar disorder. Nature.

[bib24] Mullins N., Forstner A.J., O'Connell K.S., Coombes B., Coleman J.R.I., Qiao Z., Als T.D., Bigdeli T.B., Borte S., Bryois J., Charney A.W., Drange O.K., Gandal M.J., Hagenaars S.P., Ikeda M., Kamitaki N., Kim M., Krebs K., Panagiotaropoulou G., Schilder B.M., Sloofman L.G., Steinberg S., Trubetskoy V., Winsvold B.S., Won H.H., Abramova L., Adorjan K., Agerbo E., Al Eissa M., Albani D., Alliey-Rodriguez N., Anjorin A., Antilla V., Antoniou A., Awasthi S., Baek J.H., Baekvad-Hansen M., Bass N., Bauer M., Beins E.C., Bergen S.E., Birner A., Bocker Pedersen C., Boen E., Boks M.P., Bosch R., Brum M., Brumpton B.M., Brunkhorst-Kanaan N., Budde M., Bybjerg-Grauholm J., Byerley W., Cairns M., Casas M., Cervantes P., Clarke T.K., Cruceanu C., Cuellar-Barboza A., Cunningham J., Curtis D., Czerski P.M., Dale A.M., Dalkner N., David F.S., Degenhardt F., Djurovic S., Dobbyn A.L., Douzenis A., Elvsashagen T., Escott-Price V., Ferrier I.N., Fiorentino A., Foroud T.M., Forty L., Frank J., Frei O., Freimer N.B., Frisen L., Gade K., Garnham J., Gelernter J., Giortz Pedersen M., Gizer I.R., Gordon S.D., Gordon-Smith K., Greenwood T.A., Grove J., Guzman-Parra J., Ha K., Haraldsson M., Hautzinger M., Heilbronner U., Hellgren D., Herms S., Hoffmann P., Holmans P.A., Huckins L., Jamain S., Johnson J.S., Kalman J.L., Kamatani Y., Kennedy J.L., Kittel-Schneider S., Knowles J.A., Kogevinas M., Koromina M., Kranz T.M., Kranzler H.R., Kubo M., Kupka R., Kushner S.A., Lavebratt C., Lawrence J., Leber M., Lee H.J., Lee P.H., Levy S.E., Lewis C., Liao C., Lucae S., Lundberg M., MacIntyre D.J., Magnusson S.H., Maier W., Maihofer A., Malaspina D., Maratou E., Martinsson L., Mattheisen M., McCarroll S.A., McGregor N.W., McGuffin P., McKay J.D., Medeiros H., Medland S.E., Millischer V., Montgomery G.W., Moran J.L., Morris D.W., Muhleisen T.W., O'Brien N., O'Donovan C., Olde Loohuis L.M., Oruc L., Papiol S., Pardinas A.F., Perry A., Pfennig A., Porichi E., Potash J.B., Quested D., Raj T., Rapaport M.H., DePaulo J.R., Regeer E.J., Rice J.P., Rivas F., Rivera M., Roth J., Roussos P., Ruderfer D.M., Sanchez-Mora C., Schulte E.C., Senner F., Sharp S., Shilling P.D., Sigurdsson E., Sirignano L., Slaney C., Smeland O.B., Smith D.J., Sobell J.L., Soholm Hansen C., Soler Artigas M., Spijker A.T., Stein D.J., Strauss J.S., Swiatkowska B., Terao C., Thorgeirsson T.E., Toma C., Tooney P., Tsermpini E.E., Vawter M.P., Vedder H., Walters J.T.R., Witt S.H., Xi S., Xu W., Yang J.M.K., Young A.H., Young H., Zandi P.P., Zhou H., Zillich L., Psychiatry H.A.-I., Adolfsson R., Agartz I., Alda M., Alfredsson L., Babadjanova G., Backlund L., Baune B.T., Bellivier F., Bengesser S., Berrettini W.H., Blackwood D.H.R., Boehnke M., Borglum A.D., Breen G., Carr V.J., Catts S., Corvin A., Craddock N., Dannlowski U., Dikeos D., Esko T., Etain B., Ferentinos P., Frye M., Fullerton J.M., Gawlik M., Gershon E.S., Goes F.S., Green M.J., Grigoroiu-Serbanescu M., Hauser J., Henskens F., Hillert J., Hong K.S., Hougaard D.M., Hultman C.M., Hveem K., Iwata N., Jablensky A.V., Jones I., Jones L.A., Kahn R.S., Kelsoe J.R., Kirov G., Landen M., Leboyer M., Lewis C.M., Li Q.S., Lissowska J., Lochner C., Loughland C., Martin N.G., Mathews C.A., Mayoral F., McElroy S.L., McIntosh A.M., McMahon F.J., Melle I., Michie P., Milani L., Mitchell P.B., Morken G., Mors O., Mortensen P.B., Mowry B., Muller-Myhsok B., Myers R.M., Neale B.M., Nievergelt C.M., Nordentoft M., Nothen M.M., O'Donovan M.C., Oedegaard K.J., Olsson T., Owen M.J., Paciga S.A., Pantelis C., Pato C., Pato M.T., Patrinos G.P., Perlis R.H., Posthuma D., Ramos-Quiroga J.A., Reif A., Reininghaus E.Z., Ribases M., Rietschel M., Ripke S., Rouleau G.A., Saito T., Schall U., Schalling M., Schofield P.R., Schulze T.G., Scott L.J., Scott R.J., Serretti A., Shannon Weickert C., Smoller J.W., Stefansson H., Stefansson K., Stordal E., Streit F., Sullivan P.F., Turecki G., Vaaler A.E., Vieta E., Vincent J.B., Waldman I.D., Weickert T.W., Werge T., Wray N.R., Zwart J.A., Biernacka J.M., Nurnberger J.I., Cichon S., Edenberg H.J., Stahl E.A., McQuillin A., Di Florio A., Ophoff R.A., Andreassen O.A. (2021). Genome-wide association study of more than 40,000 bipolar disorder cases provides new insights into the underlying biology. Nat. Genet..

[bib25] Ochoa E.L.M. (2022). Lithium as a neuroprotective agent for bipolar disorder: an overview. Cell. Mol. Neurobiol..

[bib26] Osete J.R., Akkouh I.A., de Assis D.R., Szabo A., Frei E., Hughes T., Smeland O.B., Steen N.E., Andreassen O.A., Djurovic S. (2021). Lithium increases mitochondrial respiration in iPSC-derived neural precursor cells from lithium responders. Mol. Psychiatr..

[bib27] Osete J.R., Akkouh I.A., de Assis D.R., Szabo A., Frei E., Hughes T., Smeland O.B., Steen N.E., Andreassen O.A., Djurovic S. (2021). Lithium increases mitochondrial respiration in iPSC-derived neural precursor cells from lithium responders. Mol. Psychiatr..

[bib28] Regenold W.T., Phatak P., Marano C.M., Sassan A., Conley R.R., Kling M.A. (2009). Elevated cerebrospinal fluid lactate concentrations in patients with bipolar disorder and schizophrenia: implications for the mitochondrial dysfunction hypothesis. Biol. Psychiatr..

[bib29] Roberts M.J., Schirra H.J., Lavin M.F., Gardiner R.A. (2011). Metabolomics: a novel approach to early and noninvasive prostate cancer detection. Kor. J. Urol..

[bib30] Rolstad S., Jakobsson J., Sellgren C., Ekman C.J., Blennow K., Zetterberg H., Palsson E., Landen M. (2015). Cognitive performance and cerebrospinal fluid biomarkers of neurodegeneration: a study of patients with bipolar disorder and healthy controls. PLoS One.

[bib31] Sachs G.S., Thase M.E., Otto M.W., Bauer M., Miklowitz D., Wisniewski S.R., Lavori P., Lebowitz B., Rudorfer M., Frank E., Nierenberg A.A., Fava M., Bowden C., Ketter T., Marangell L., Calabrese J., Kupfer D., Rosenbaum J.F. (2003). Rationale, design, and methods of the systematic treatment enhancement program for bipolar disorder (STEP-BD). Biol. Psychiatr..

[bib32] Scola G., Andreazza A.C., Young L.T. (2018). A comparative expression analysis of isocitrate dehydrogenase-3 gene and protein levels in postmortem brain tissues from subjects with bipolar disorder. Mol. Psychiatr..

[bib33] Scola G., Kim H.K., Young L.T., Andreazza A.C. (2013). A fresh look at complex I in microarray data: clues to understanding disease-specific mitochondrial alterations in bipolar disorder. Biol. Psychiatr..

[bib34] Stern S., Santos R., Marchetto M.C., Mendes A.P.D., Rouleau G.A., Biesmans S., Wang Q.W., Yao J., Charnay P., Bang A.G., Alda M., Gage F.H. (2018). Neurons derived from patients with bipolar disorder divide into intrinsically different sub-populations of neurons, predicting the patients' responsiveness to lithium. Mol. Psychiatr..

[bib35] Stork C., Renshaw P.F. (2005). Mitochondrial dysfunction in bipolar disorder: evidence from magnetic resonance spectroscopy research. Mol. Psychiatr..

[bib36] Sun X., Wang J.F., Tseng M., Young L.T. (2006). Downregulation in components of the mitochondrial electron transport chain in the postmortem frontal cortex of subjects with bipolar disorder. J. Psychiatry Neurosci..

[bib37] Wellen K.E., Hatzivassiliou G., Sachdeva U.M., Bui T.V., Cross J.R., Thompson C.B. (2009). ATP-citrate lyase links cellular metabolism to histone acetylation. Science.

[bib38] Yoshimi N., Futamura T., Bergen S.E., Iwayama Y., Ishima T., Sellgren C., Ekman C.J., Jakobsson J., Palsson E., Kakumoto K., Ohgi Y., Yoshikawa T., Landen M., Hashimoto K. (2016). Cerebrospinal fluid metabolomics identifies a key role of isocitrate dehydrogenase in bipolar disorder: evidence in support of mitochondrial dysfunction hypothesis. Mol. Psychiatr..

[bib39] Yoshimi N., Futamura T., Kakumoto K., Salehi A.M., Sellgren C.M., Holmen-Larsson J., Jakobsson J., Palsson E., Landen M., Hashimoto K. (2016). Blood metabolomics analysis identifies abnormalities in the citric acid cycle, urea cycle, and amino acid metabolism in bipolar disorder. BBA Clin..

[bib40] Zverova M., Hroudova J., Fisar Z., Hansikova H., Kalisova L., Kitzlerova E., Lambertova A., Raboch J. (2019). Disturbances of mitochondrial parameters to distinguish patients with depressive episode of bipolar disorder and major depressive disorder. Neuropsychiatric Dis. Treat..

